# Effects of sorghum varieties on microbial communities and volatile compounds in the fermentation of light-flavor Baijiu

**DOI:** 10.3389/fmicb.2024.1421928

**Published:** 2024-07-31

**Authors:** Jie Tang, Bin Lin, Yimin Shan, Song Ruan, Wei Jiang, Qun Li, Liping Zhu, Rui Li, Qiang Yang, Hai Du, Shengzhi Yang, Qi Sun, Shenxi Chen

**Affiliations:** ^1^Hubei Key Laboratory of Quality and Safety of Traditional Chinese Medicine Health Food, Jing Brand Research Institute, Jing Brand Co., Ltd., Daye, China; ^2^Lab of Brewing Microbiology and Applied Enzymology, Key Laboratory of Industrial Biotechnology of Ministry of Education, School of Biotechnology, Jiangnan University, Wuxi, China

**Keywords:** light-flavor Baijiu, sorghum varieties, fermented grains, microbial community, volatile compounds

## Abstract

Light-flavor Baijiu (LFB) fermentation is a representative spontaneous mixed-culture solid-state fermentation process in which sorghum is used as the raw material. Raw materials and microorganisms are crucial to the flavor formation and quality of LFB. However, the microbial and physicochemical dynamics of different sorghum varieties during LFB fermentation, as well as their impact on flavor compounds are still largely unknown. Herein, PacBio single-molecule real-time (SMRT) sequencing and headspace solid-phase microextraction coupled with gas chromatography–mass spectrometry (HS-SPME-GC–MS) were applied to investigate microbial community succession and volatile flavor formation in glutinous/non-glutinous sorghum-based fermented grains during LFB fermentation. Fermented grains made of glutinous sorghum Liangnuo No. 1 (GLN) had higher bacterial α-diversity and lower fungal α-diversity than those with fermented grains prepared with non-glutinous red sorghum (NRS) (*p* < 0.05). The dominant microbial species were *Saccharomyces cerevisiae*, *Acetobacter pasteurinus*, and *Lactobacillus helveticus*, the latter two of which were the predominant bacteria observed at the end of fermentation in GLN and NRS, respectively. Moisture content and reducing sugar had a more significant impact on the microorganisms in GLN, while amino acid nitrogen, total free amino acids, and residual starch were the main driving factors driving the microbial community in NRS. The correlation network and discriminant analysis indicated that a relatively high content of 4-vinylguaiacol showed a significant positive association with significant differential microbial species in GLN. These results provided valuable insights for improving the quality of LFB.

## Introduction

Baijiu (Chinese liquor), which is one of the six well-known distilled spirits throughout the world, plays an indispensable role in Chinese culture, economy, and dietary profiles (Jin et al., 2017; Wang, 2022). Based on its unique taste and characteristic flavor profile, Baijiu can be divided into four basic categories: sauce-flavor Baijiu, strong-flavor Baijiu, light-flavor Baijiu, and rice-flavor Baijiu (Ye et al., 2021). Light-favor Baijiu (LFB) is a type of Chinese liquor with a pure and mild flavor produced by traditional spontaneous mixed-culture solid-state fermentation. Unlike other types of Baijiu, traditional LFB is fermented in ceramic jars and mainly includes Jiuqu (starter) preparation, material pretreatment, alcoholic fermentation, distillation, and aging (Pang et al., 2020). The flavor components of Baijiu are subjected to complicated interactions among several factors during the whole process, involving the properties and processing of the raw material, substances produced during fermentation, microbial metabolites from starter and fermented containers, characteristics of the environmental microbiota, and distillation of fermented grains (Pang et al., 2020; Wu et al., 2021). Thus, raw materials and microbial metabolites are of great importance to the formation of Baijiu flavor.

As the principal raw material in the manufacturing of LFB, sorghum is rich in starch and protein and contains small amounts of tannin and fiber, which influence microbial communities and the flavor profile of fermented grains (Xu et al., 2018; Chen et al., 2019; Liu C. et al., 2021; Wang et al., 2021). According to the ratio of amylopectin to amylose in the grain, sorghum varieties can be classified as glutinous or non-glutinous, and the amylopectin/amylose ratio for common sorghum is approximately about 80/20, whereas in glutinous sorghum, there is little or no amylose (Chen et al., 2019). Compared to that in non-glutinous sorghum, a high ratio of amylopectin to amylose in glutinous sorghum can more effectively convert starch into ethanol (Wang et al., 2008). However, the yield of glutinous sorghum is relatively low, and the cost of using these grains for Baijiu production is greater. The selection of raw materials may affect the quality and value of Baijiu. To clarify the effects of sorghum varieties on the quality of Baijiu, different types of sorghum and microbial community association in Chinese strong-flavor and Xifeng Baijiu fermentation systems have been examined *in situ* (Liu C. et al., 2021; Liu et al., 2023). However, to our knowledge, the relationships between sorghum varieties, microbial communities and volatile compounds during LFB fermentation have not yet been reported.

The flavor of Baijiu is the foremost factor in determining its quality and is formed by microbial community under the driving force of various environmental factors, such as moisture, temperature, and acidity (Zhang et al., 2020; Ji et al., 2023; Wang et al., 2023). Hence, the exploration of the associations among the microbial community, environmental factors, and flavor compounds has become the key to clarify the mechanism underlying the formation of Baijiu flavor. Recent studies on LFB have mainly focused on the elucidation of microbial community and their correlation with flavor compound formation (Luo et al., 2023; Pan et al., 2023), as well as correlational analyses of physicochemical properties, microbial communities, and volatile components in Jiuqu (Hu et al., 2023; Yu et al., 2023) and comparative analyses of the microbial community structure and screening of functional microbial strains (Tang et al., 2022a; Xiang et al., 2023). However, the dynamics of the environmental factors, microbial community, and flavor compounds in fermented grains are still not fully understood. In particular, the impact of sorghum varieties on microbial communities and flavor compounds has rarely been considered.

The fermented grain samples that were used in this study were collected from Hubei Province and fermented by glutinous sorghum and non-glutinous sorghum with Jiuqu, respectively. Headspace solid-phase microextraction coupled with gas chromatography–mass spectrometry (HS-SPME-GC–MS) and PacBio single-molecule real-time (SMRT) sequencing were applied to determine the volatile compounds and microbial community structure during different stages of LFB fermentation, respectively. Moreover, the driving effect of environmental factors on microbial communities and the correlation between microbiota and the volatile flavor profiles were investigated. The results can provide a theoretical basis for elucidating the brewing mechanism and improving the quality and fermentation efficiency of LFB.

## Materials and methods

### Sample collection and treatment

Fermented grain samples were collected in July 2022 from Jing Brand Co., Ltd., a LFB producer in Huangshi, Hubei Province, China, which has an annual Baijiu production of 70,000 tons. The mechanized technology for the brewing process of LFB was showed in detail in our previous study (Tang et al., 2022b). Two sorghum varieties were selected: glutinous sorghum Liangnuo No. 1 (GLN) and non-glutinous red sorghum (NRS). Fermented grain samples (250 g) were collected on days 0, 1, 2, 3, 4, 5, 7, 9, 11, and 14 during fermentation from fermentation tanks in the middle layer, and labeled as GD0, GD1, GD2, GD3, GD4, GD5, GD7, GD9, GD11, GD14 (fermenting with GLN), and JD0, JD1, JD2, JD3, JD4, JD5, JD7, JD9, JD11, JD14 (fermenting with NRS), respectively. These samples were stored at 4°C for physicochemical properties analysis, and − 80°C for DNA extraction and volatile compounds analysis. Three independent batches were sampled for adequate representation. A total of 60 samples were collected for analysis.

### Physicochemical analysis

Moisture was measured by estimating the weight loss using drying fermented grain samples (10 g) to a constant weight at 110°C for 3 h. Acidity, amino acid nitrogen, and reducing sugar were detected as described by Lin et al. (2022). Direct titration was used to determine the acidity and amino acid nitrogen of fermented grains. Reducing sugar was detected by the DNS method. Hydrochloric acid dissolution was used to determine residual starch content (Tang et al., 2022b). The determination of free amino acids content referred to“GB5009.124–2016 Determination of Free amino acids in Foods.” All physicochemical analyses were conducted in triplicate.

### HS-SPME-GC–MS analysis of volatile compounds

The volatile compounds in fermented grains were determined by HS-SPME-GC–MS according to the protocol described previously (Sun et al., 2022). Concretely, 10 g of each fermented grain sample was added to 25 mL of sterilized ultrapure water and ultrasonicated for 30 min, and then soaked overnight at 4°C. The suspension was centrifuged at 8000 × g at 4°C for 10 min. 8 mL of the supernatant was transferred to a 20 mL headspace vial containing 20 μL of internal standard mixer (ethyl caproate-d11, hexanal-d12, n-hexanol-d13, and ethyl octanoate-d15) and 7 g of sodium chloride. The headspace vial was placed in the automatic microextraction device and extracted at 50°C for 45 min. A total of 60 fermented grain samples were collected, followed by GC–MS analysis. Volatile compounds were identified by matching with the National Institute of Standards and Technology (NIST) library (Gaithersburg, MD, United States), and the matching masses were more than 80%. The semi-quantification of the volatiles was determined with the internal standard method. The relative concentration of volatile compounds was calculated based on the ratio of volatiles’ peak area and internal standard peak area.

### DNA extraction, amplification, and sequencing

Total genomic DNA from the fermented grain samples was extracted by using the TGuide S96 Magnetic Soil/Stool DNA Kit (Tiangen Biotech (Beijing) Co., Ltd.) according to the manufacturer’s instructions. The quality and quantity of the extracted DNA were examined via electrophoresis on a 1.8% agarose gel, and the DNA concentration and purity were determined with a NanoDrop 2000 UV–Vis spectrophotometer (Thermo Scientific, Wilmington, United States). The full-length 16S rRNA gene in bacteria was amplified with the primer pairs 16S-F (5′-AGRGTTTGATYNTGGCTCAG-3′) and 16S-R (5′-TASGGHTACCTTGTTASGACTT-3′) (Johnson et al., 2019). The primers for fungal analysis were designed on the basis of the full-length internal transcribed spacer (ITS) regions of the rRNA (ITS1F: 5′-CTTGGTCATTTAGAGGAAGTAA-3′; ITS4: 5′-TCCTCCGCTTATTGATATGC-3′) (Banerjee et al., 2019). All PCR reactions were performed in a 30-μL reaction system. The KOD One PCR Master Mix containing KOD DNA polymerase (TOYOBOLife Science) was used to perform PCR amplification. The PCR conditions for 16S rRNA region amplification were as follows: initial denaturation at 95°C for 2 min, followed by 25 cycles of denaturation at 98°C for 10 s, annealing at 55°C for 30 s, and extension at 72°C for 1 min 30 s, and a final step at 72°C for 2 min. The PCR conditions for fungal full-length ITS amplification were as follows: pre-denaturation at 95°C for 2 min, 32 cycles of denaturation at 98°C for 30 s, annealing at 55°C for 30 s, and extension at 72°C for 45 s, as well as a final extension at 72°C for 5 min. The amplicons were quantified, after which the normalized equimolar concentrations of amplicons were pooled and sequenced on the PacBio Sequel II platform (Allwegene Tech., Beijing, China).

### Bioinformatics and statistical analysis

Raw circular consensus sequencing (CCS) was performed by identifying CCS reads through barcodes via the Lima v1.7.0 software. Cutadapt v2.7 was applied to identify and remove primer sequences and acquire clean CCS sequences by filtering sequence length. Subsequently, effective CCS sequences were obtained by identifying and removing chimeric sequences via UCHIME v4.2 (Lai et al., 2023). The qualified sequences with more than 97% similarity thresholds were clustered into operational taxonomic units (OTUs) by using USEARCH v10.0 (Robert, 2013). The Naive Bayes classifier in QIIME2 using the SILVA database (release 138.1) with a confidence threshold of 70% was used to annotate the prokaryotic OTUs (Bolyen et al., 2019). Fungal OTUs were annotated with the fungal ITS database, UNITE (Release 8.0) as the reference sequence database. Microbial α-diversity indices (Chao 1 richness estimator and Shannon’s diversity index) were calculated to determine the complexity of the species diversity of each sample utilizing QIIME2 software. Additionally, we employed linear discriminant analysis (LDA) effect size (LEfSe) to evaluate the differentially abundant taxa among the groups, and the threshold for discriminative features was a logarithmic LDA score of 4.0 (Segata et al., 2011).

The dynamic changes in physicochemical indicators, microbial α-diversity indices, and relative abundance of bacteria and fungi in the samples were plotted using OriginPro 2018 software (Origin Lab Corporation, United States). Advanced Circos barplot, heatmap plots, PLS-DA (partial least squares-discriminant analysis), STAMP (statistical analysis of metagenomic profiles), heatmap barplot, Procrustes analysis graph, and linear regression plot were performed using the OmicStudio tools at https://www.omicstudio.cn/tool. Redundancy analysis (RDA) performed using the vegan packages in R was chose to determine the correlations between physicochemical properties and microbial community and the Monte Carlo permutation test was used to check the significance (Pan et al., 2022). To explore the correlations between the dominant microbiota and important aroma components, Spearman’s correlation coefficient (ρ) was explored between microorganisms and metabolites via IBM SPSS Statistics (version 19.0), and visualized as a correlation network with |ρ| > 0.6 and *p* < 0.05 in Cytoscape (v3.9.1) (Lin et al., 2022). The correlations between physicochemical properties and dominant microbial species were calculated by Spearman’s correlations (Huang et al., 2018; Luo et al., 2023).

All the experiments for physiochemical properties determination were conducted in triplicate. Data were presented as mean values ± standard deviation. One-way ANOVA analysis and the multiple comparisons were performed using Duncan’s test by SPSS software (version19.0, Chicago, IL, United States).

## Results

### Physicochemical properties of fermented grains

**Figure 1:**
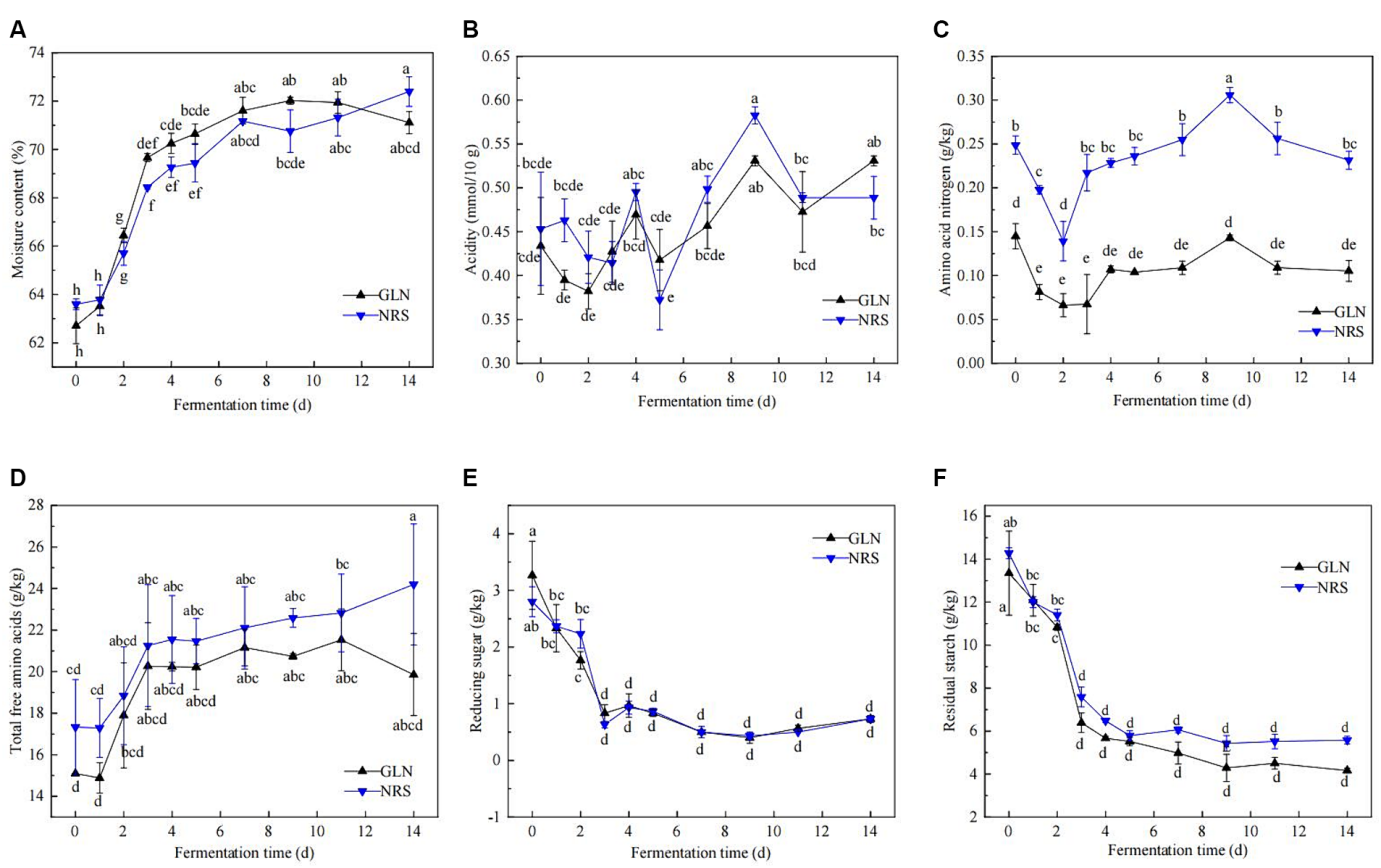
Dynamic changes of physicochemical properties during the fermentation process using two types of sorghum. (**A**) Moisture content. (**B**) Acidity. (**C**) Amino acid nitrogen. (**D**) Total free amino acids. **(E)** Reducing sugar. **(F)** Residual starch. GLN: Representing the samples fermented using glutinous Liangnuo No.1. NRS: Representing the samples fermented using non-glutinous red sorghum. Different lowercase letters indicate significant differences at the 0.05 level. The same below.

The physicochemical properties of fermented grains, including the moisture content, acidity, amino acid nitrogen content, reducing sugar, residual starch, and total free amino acids, were determined. Figure 1 illustrated that the dynamics of the physicochemical properties throughout the fermentation process of the two types of sorghum exhibited similar trends. The moisture content was approximately 63.0% at the beginning of fermentation, quickly increased to 68.4–70.0% on day 3, and then moderately escalated to approximately 72.0% thereafter (Figure 1A). The moisture content in GLN were generally higher than that in NRS (Supplementary Figure S1A). The titratable acidity rapidly decreased from 0.43 to 0.38 mmol/10 g from day 0 to day 2 in GLN, while the acidity in NRS witnessed a slight drop in the first 3 days and then gradually increased in the later stage (Figure 1B). Throughout fermentation, the acidity in GLN was often lower than that in NRS (Supplementary Figure S1B). Figures 1B,C showed that the dynamics of amino acid nitrogen and acidity in GLN and NRS were similar, and amino acid nitrogen content in GLN were markedly lower than that in NRS (*p* < 0.001) (Supplementary Figure S1C). During fermentation, the trend of the total free amino acids content curve was similar to that of the moisture content curve (Figure 1D). And the total free amino acids content in GLN was significantly lower than that in NRS (*p* < 0.05) (Supplementary Figure S1D). As shown in Figures 1D,F, the variations in reducing sugar were consistent with those in residual starch. Specifically, both of them rapidly decreased from day 0 to day 3, and then remained largely stable in the later stage, while GLN had high reducing sugar and low residual starch contents compared to NRS (Supplementary Figures S1E,F).

### Microbial diversity and structure of microbial communities

**Figure 2:**
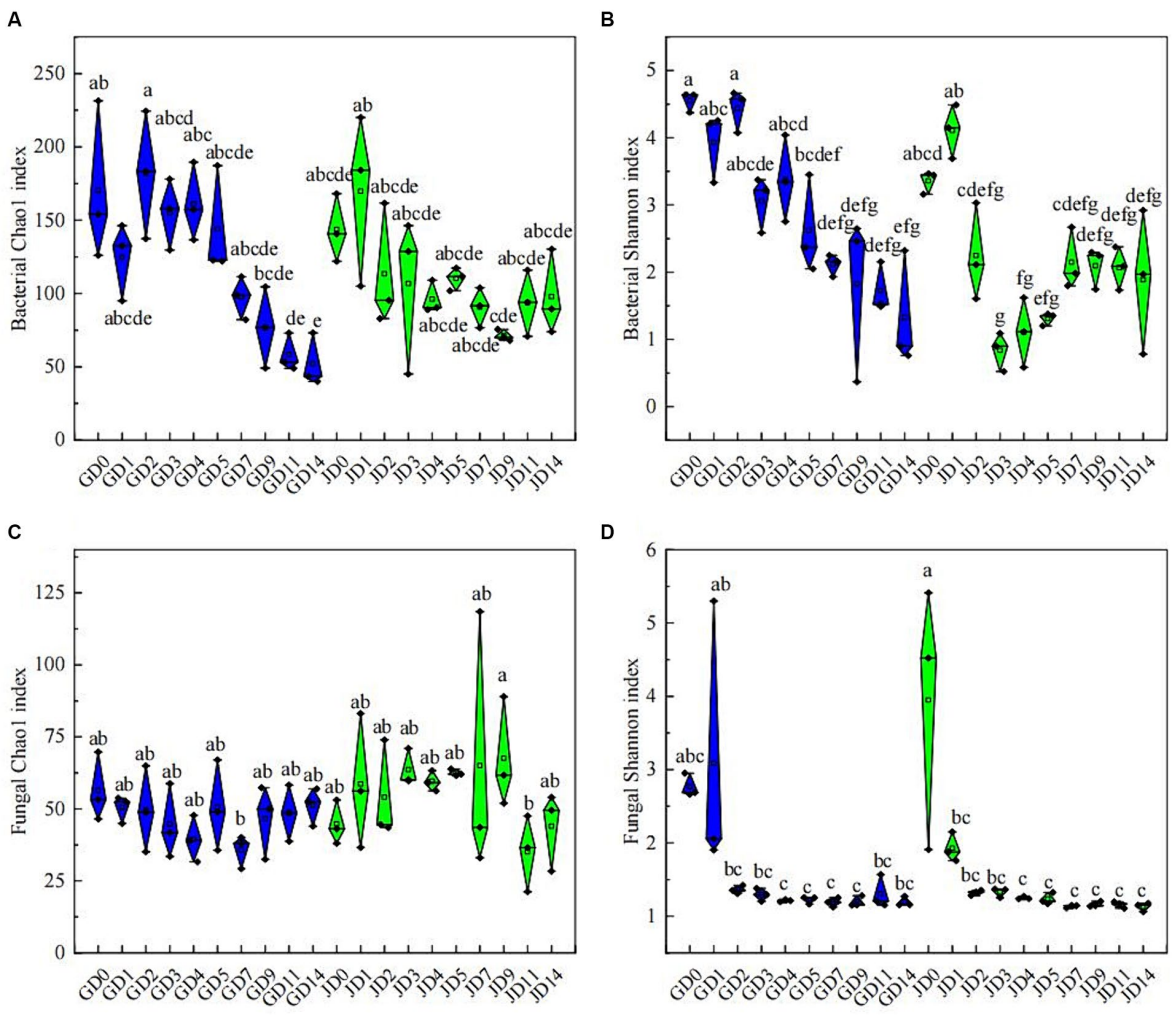
Microbial community α-diversity indices in fermented grains. **(A)** Bacterial Chao 1 indices, **(B)** bacterial Shannon indices, **(C)** fungal Chao 1 indices and **(D)** fungal Shannon indices at different fermentation times.

Following quality control, 758,105 and 2,502,927 effective CCS sequences for bacteria and fungi, respectively, were found in all samples. All of the rarefaction curves of the tested species became saturated, thus demonstrating the effectiveness of the sequencing data (Supplementary Figure S2). Changes in microbial diversity were investigated according to PacBio SMRT sequencing data. Throughout the fermentation using two types of sorghum, the α-diversity of bacteria exhibited a clear decreasing trend, especially in the later stage (Figures 2A,B). Overall, the bacterial α-diversity in GLN was higher than that in NRS, particularly for bacteria, which had a higher Shannon index in GLN (*p* < 0.05) (Supplementary Figure S3B). For fungi, the Chao 1 index showed no significant change during fermentation, while the Shannon index reached a maximum value at the beginning of fermentation and then decreased and remained stable (Figures 2C,D). Overall, NRS had a higher fungal Chao 1 index than that in GLN (*p* < 0.05) (Supplementary Figure S3C).

**Figure 3:**
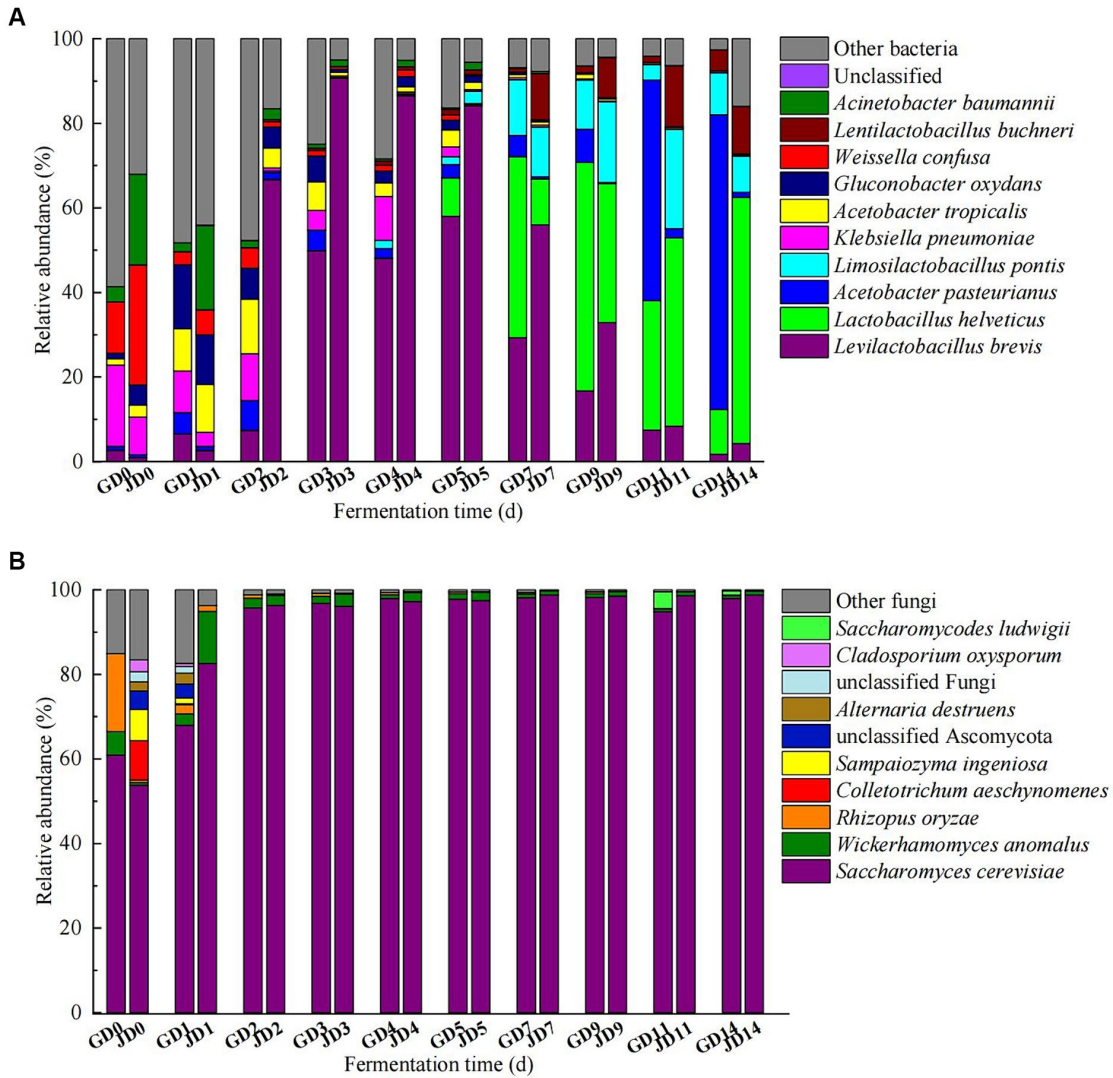
Dynamic changes of bacterial **(A)** and fungal **(B)** communities at the species level in fermented grains.

The top 10 microorganisms in the rankings dominated the microbial community during the fermentation (most of them with average relative abundances >1%). The top 10 bacteria included *Levilactobacillus brevis* (1.75–58.01% in GLN, 0.92–90.71% in NRS), *Lactobacillus helveticus* (0.04–54.04% in GLN, 0.01–58.17% in NRS), *Acetobacter pasteurinus* (0.87–69.68% in GLN, 0.07–2.18% in NRS), *Limosilicobacillus pontis* (0.02–13.17% in GLN, 0.002–23.51% in NRS), *Klebsiella pneumoniae* (0.02–19.22% in GLN, 0.04–8.94% in NRS), *Acetobacter tropicalis* (0.05–10.00% in GLN, 0.30–11.32% in NRS), *Gluconobacter oxydans* (0.00–15.13% in GLN, 0.12–11.72% in NRS), *Weissella confusa* (0.00–12.11% in GLN, 0.003–28.35% in NRS), *Lentilactobacillus buchneri* (0.02–5.00% in GLN, 0.02–14.44% in NRS), and *Acinetobacter baumannii* (0.00–3.62% in GLN, 0.05–21.45% in NRS) during fermentation. On day 0 and 1, the most common bacteria were *A. baumannii*, *W. confusa*, *G. oxydans*, *A. tropicalis*, and *K. pneumoniae.* As fermentation progressed, acetic acid bacteria and lactic acid bacteria dominated in the middle and later phases of fermentation. The relative abundance of *La. brevis* first increased and then decreased, and that for GLN was lower than that for NRS. *La. helveticus* became the dominant species after 5 days and rapidly increased in the later stage, and it predominated (58.17%) at the end of fermentation in NRS, which was higher than that in GLN. However, *A. pasteurinus* became the dominant specie (69.68%) at the end of fermentation in GLN, and its relative abundance was higher than that in NRS (Figure 3A).

The most common fungal species were *Saccharomyces cerevisiae*, *Wickerhamomyces anomalus*, and *Rhizopus oryzae*. Throughout the entire fermentation, *S. cerevisiae* was the dominant yeast, and its relative abundance reached over 95% after day 1. The relative abundance of *S. cerevisiae* on day 0 in GLN was higher than that in NRS; however, the proliferation rate of *S. cerevisiae* in NRS (51.73–96.31%) was greater than that in GLN (60.94–95.70%) from day 0 to day 2 (Figure 3B).

**Figure 4:**
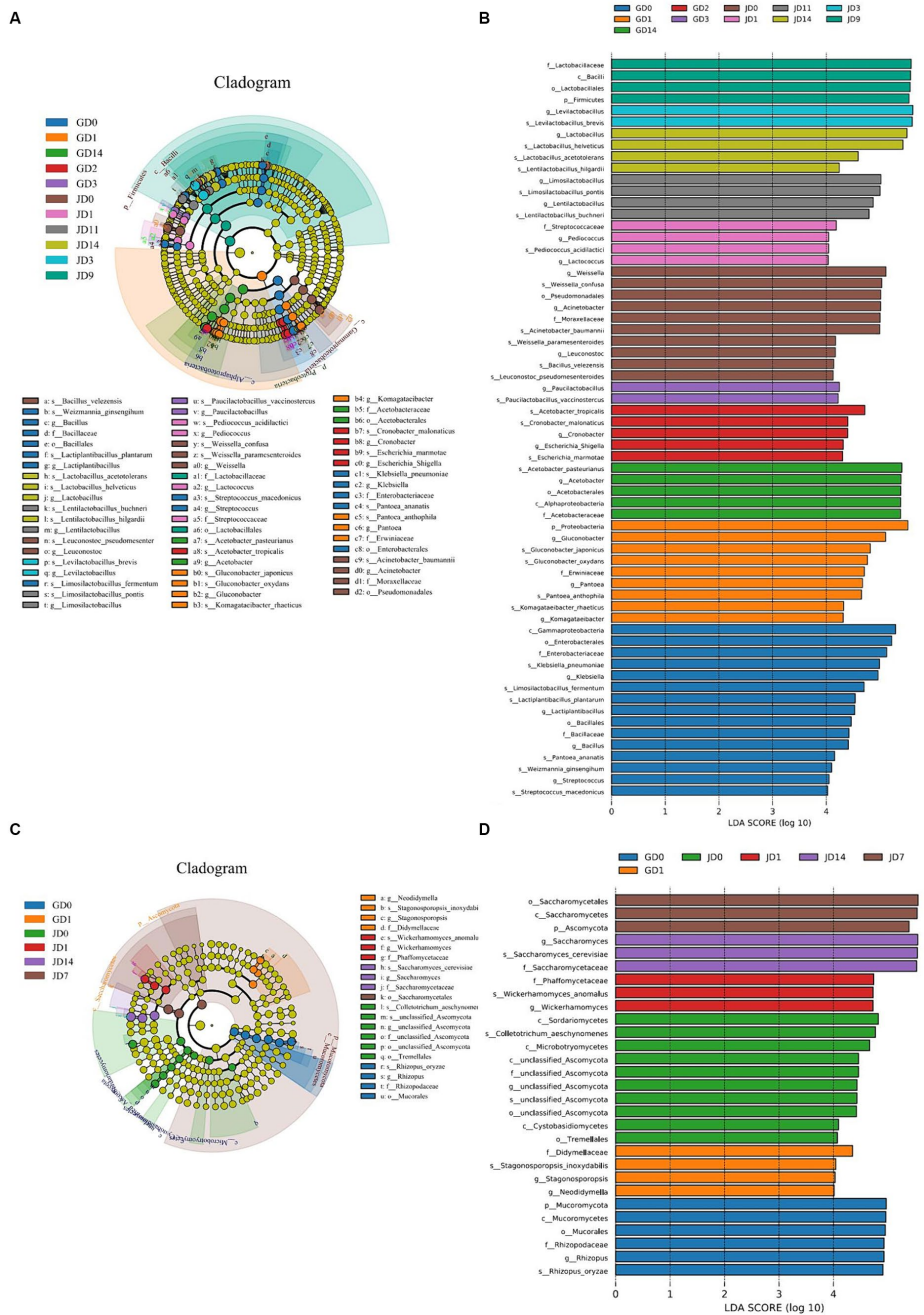
LEfSe analysis of inter group samples during fermentation process. **(A)** Bacterial and **(C)** fungal taxonomic cladogram of the discriminant analyzed by LEfSe. Taxonomic ranks from phylum to genus were represented by rings from the inner to the outer portion of the graph. The nodes in each classification were defined at the same level of taxonomic rank and the sizes of the nodes were proportional to their relative abundances. Nodes with lime color indicated no significant variation in the abundances of the taxa. Highlighted areas with the additional different colors implied different groups, and the nodes in the extra different colors distinguished the different samples. Nodes with the same color in the branches were defined as significantly different taxonomic biomarkers representing the different taxa in the same color group. (**B**) Bacterial and **(D)** fungal taxa that showed significantly different abundances for samples.

The linear discriminant analysis (LDA) effect size (LEfSe) method was also used to analyze the differences in the microbial taxa detected in all of the samples. For bacteria, LDA highlighted that a total of 64 biomarkers were identified via the statistically significant LDA threshold of >4 (Figure 4A). A phylogenetic tree of the total bacterial community from the phylum to the species level was constructed for these differentiating taxa by using LEfSe (Figure 4B). A total of 2 phyla, 3 classes, 5 orders, 7 families, 20 genera, and 27 species displayed significant differences in abundance among all of the samples. The 27 bacterial species with significant differences included *K. pneumoniae* (6.06% in GLN, 1.48% in NRS), *Limosilactobacillus fermentum* (3.40% in GLN, 0.01% in NRS), *Lactiplantibacillus plantarum* (2.82% in GLN, 0.01% in NRS), *G. oxydans* (3.50% in GLN, 2.70% in NRS), *Gluconobacter japonicus* (2.45% in GLN, 1.13% in NRS), *A. pasteurianus* (14.39% in GLN, 0.74% in NRS), *A. tropicalis* (4.02% in GLN, 2.58% in NRS), *W. confusa* (2.31% in GLN, 3.73% in NRS), *Bacillus velezensis* (0.59% in GLN, 0.92% in NRS), *A. baumannii* (0.93% in GLN, 4.82% in NRS), *La. buchneri* (1.57% in GLN, 5.01% in NRS), *La. pontis* (4.99% in GLN, 6.88% in NRS), *Lentilactobacillus hilgardii* (0.12% in GLN, 0.83% in NRS), *La. acetotolerans* (0.30% in GLN, 1.22% in NRS), *La. helveticus* (12.90% in GLN, 14.83% in NRS), and *La. brevis* (22.62% in GLN, 43.12% in NRS), etc. Among them, 15 and 12 species were distributed in the fermented grains with GLN and NRS, respectively.

For fungi, 29 biomarkers, which included 2 phyla, 6 classes, 4 orders, 5 families, 6 genera, and 6 species, were detected in all of the samples (Figure 4D). The six fungal species were *R. oryzae*, *S. cerevisiae*, *W. anomalus*, *C. aeschynomenes*, *Stagonosporopsis inoxydabilis*, and unclassified Ascomycota. More differential fungal species were detected in the NRS-treated fermented grains.

### Characteristics of volatile flavor compounds

**Figure 5:**
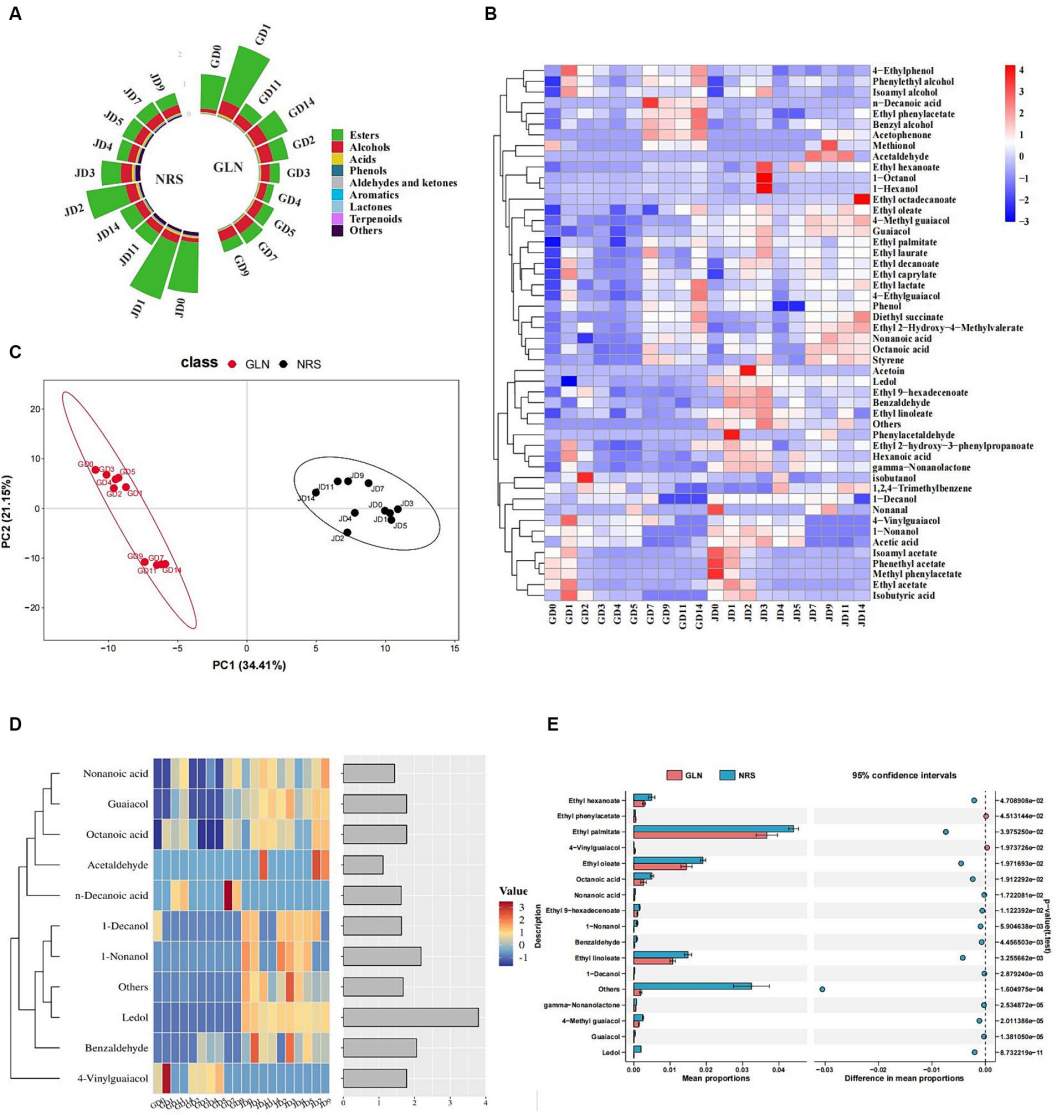
The key volatile substances composition in GLN and NRS. **(A)** The content of volatile flavor compounds in GLN and NRS. **(B)** Heatmap of flavor components in GLN and NRS. **(C)** PLS-DA between volatile compounds in 20 types of samples in GLN and NRS. **(D)** A heatmap and classification of 11 volatile compounds with the variable importance (VIP) value >1.0 based on the PLS-DA of GLN and NRS. **(E)** STAMP analysis of significant differential flavor compounds in GLN and NRS.

Approximately 59 and 65 major volatile compounds in the fermented grains prepared with GLN and NRS, respectively, were identified by HS-SPME-GC–MS. All of the volatiles were composed of nine categories, including esters, alcohols (excluding ethanol), acids, phenols, aldehydes and ketones, aromatics, lactones, terpenoids, and others. Figure 5A showed that esters, which were the most abundant volatiles in the early stage, followed by alcohols and acids, had the highest content in both GLN and NRS. The relative content of esters reached its maximum on day 1, and rapidly decreased from day 1 to day 3, and then remained relatively stable. The contents of esters (average content 0.64 mg/kg) in GLN were lower than those (average content 0.82 mg/kg) in NRS. Alcohols rapidly increased from day 0 (0.12 mg/kg-0.14 mg/kg) to day 1 (0.28 mg/kg-0.45 mg/kg) and then remained relatively stable (0.30 mg/kg-0.40 mg/kg). The contents of alcohols (average content 0.33 mg/kg) in GLN were higher than those (average content 0.28 mg/kg) in NRS. The change in acids in GLN showed a clear trend with respect to the ester content, whereas the acid content in NRS was greatest (0.075 mg/kg) on day 3 and then decreased.

Heatmap analysis of 49 flavor components in the fermented grains, including 18 esters, 9 alcohols, 6 acids, 6 phenols, 6 aldehydes and ketones, 2 aromatic compounds, 1 lactone, and 1 terpenoid, which suggested that the relative content of volatiles in NRS was relatively higher than that in GLN (Figure 5B). The 15 main flavor components with relative contents greater than 1% included ethyl acetate, phenethyl acetate, ethyl palmitate, isoamyl acetate, ethyl oleate, ethyl linoleate, ethyl decanoate, ethyl caprylate, diethyl succinate, phenylethyl alcohol, isobutanol, isoamyl alcohol, and acetic acid, etc. Ethyl acetate was the most abundant ester in the early stage and then decreased and remained relatively stable in the later stage. In addition to ethanol, phenylethyl alcohol was the most abundant alcohol, which showed a rapid increase from day 0 to day 3 and then remained relatively stable. The change in isoamyl alcohol content exhibited a similar trend to that of phenylethyl alcohol. The contents of phenylethyl alcohol and isoamyl alcohol in GLN were higher than those in NRS. Acetic acid was the most abundant acid and its content in GLN was lower than that in NRS.

All of the volatiles were split into 20 groups for PLS-DA to illustrate the grouping of GLN and NRS, flavor compounds, and notable distinctive substances. The *R*^2^ (0.98) and *Q*^2^ (0.95) values implied that the established model was valid. PLS-DA demonstrated that sorghum variety had a significant effect on the flavor profiles of the fermented grains. In addition, the flavor structures of the samples from day 0 to day 5 were relatively similar, and those of the samples from day 7 to day 14 were clustered together (Figure 5C). The fermentation could be divided into two stages (stage 1: day 0 to day 5; stage 2: day 7 to day 14) based on the composition of flavor substances in GLN, whereas the flavor substances varied slightly in NRS, thus resulting in no significant difference during fermentation. Flavor substances with variable importance in the projection (VIP) > 1.0 are usually used to explain the clustering of diverse groups in discriminant analysis. Eleven distinctive compounds with VIP > 1.0 were identified by using PLS-DA, including 2 alcohols, 3 acids, 2 aldehydes, 2 phenols, 1 terpenoid, and 1 other (Figure 5D). STAMP was applied to compare the differences in flavor substances between GLN and NRS to obtain significantly different flavor compounds. Thirteen distinctive compounds were identified via STAMP, including 6 esters, 1 aldehyde, 3 phenolics, 1 lactone, 1 terpenoid, and 1 other (Figure 5E). Among them, 4-vinylguaiacol and n-decanoic acid were more abundant in GLN (Figure 5D).

### Abiotic factors driving microbial community succession

**Figure 6:**
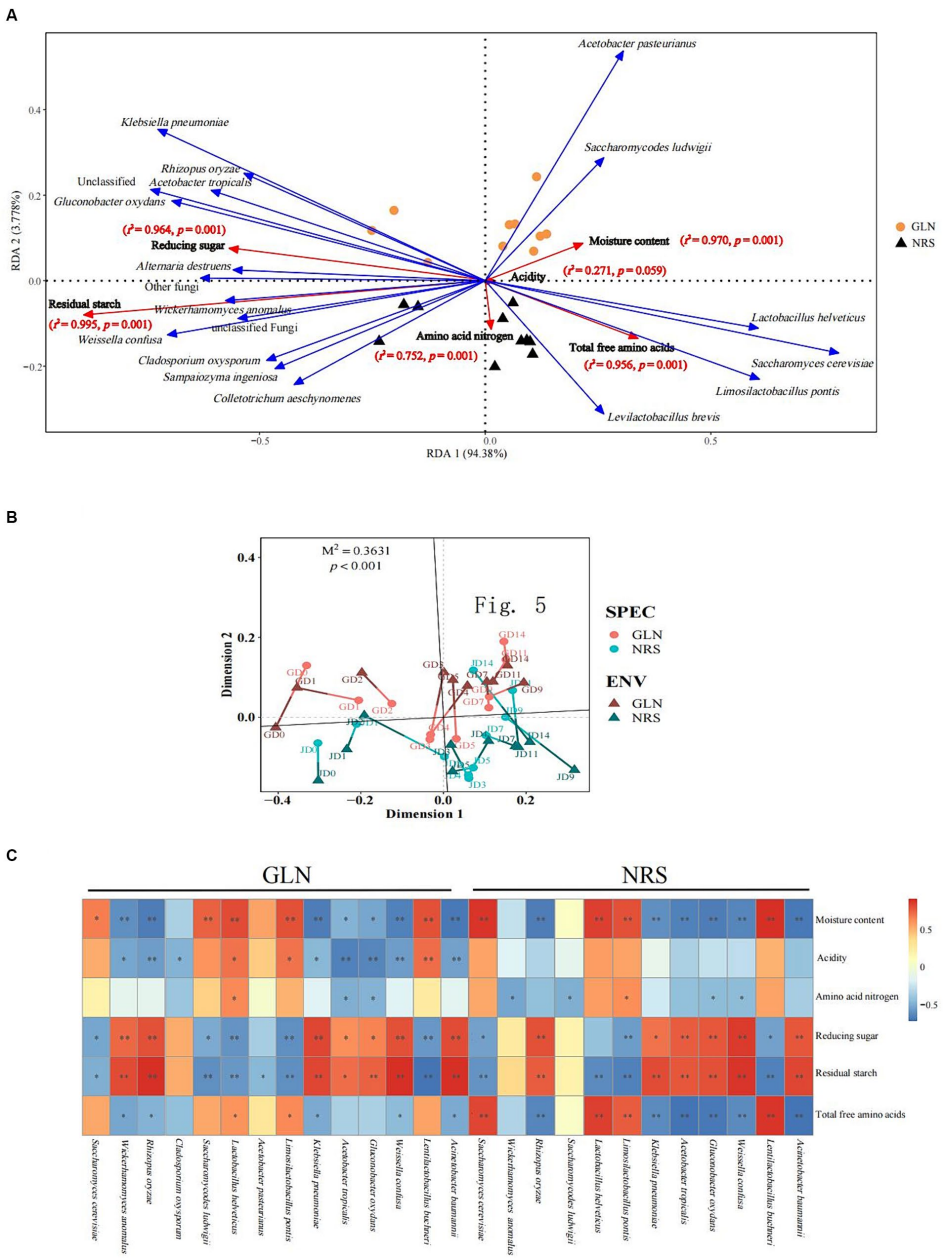
The driving force of microbial community succession. **(A)** Redundancy analysis of microbial and abiotic factors in fermented grains. **(B)** Procrustes analysis of the correlation between microbial community and abiotic factors in fermented grains (M^2^ = 0.3631, *p* < 0.001, 999 permutations). **(C)** Correlation of microorganisms and physicochemical properties (Spearman’s |ρ| > 0.6 and *p* < 0.05).

The RDA results showed that the two axes explained 98.16% of the total variation in the microbial community (Figure 6A). The Monte Carlo permutation test results showed that moisture and reducing sugar both had a more significant impact on microorganisms in GLN than those in NRS. However, amino acid nitrogen, total free amino acids, and residual starch were more important for microorganisms in NRS. As shown in Figure 6B, on the basis of the NMDS analysis of both microbial abundance and environmental factors, the results of Procrustes analysis indicated that there was a significant correlation between the microbial community and environmental factors in different samples (M^2^ = 0.3631, *p* < 0.001).

Additionally, to better understand the impact of abiotic factors on microorganisms, Spearman correlation coefficients were used to examine the correlations between physicochemical properties and dominant microbial species (Figure 6C). There was a significant correlation between moisture content, reducing sugar, residual starch and a considerable fraction of fungi and bacteria. *S. cerevisiae*, *La. buchneri*, and *La. pontis* were positively correlated with moisture content and negatively correlated with reducing sugar and residual starch, while *R. oryzae*, *K. pneumoniae*, *A. tropicalis*, *G. oxydans*, *W. confusa*, and *A.baumannii* were negatively related to moisture content and positively related to reducing sugar and residual starch (Supplementary Tables S2, S3). Compared to those in NRS, more microbial species in GLN were driven by physicochemical properties. For instance, acidity was only significantly correlated with microbial species in GLN, thus indicating the importance of acidity to the microbial community in GLN.

### Relationships between major flavor components and microbial species

**Figure 7:**
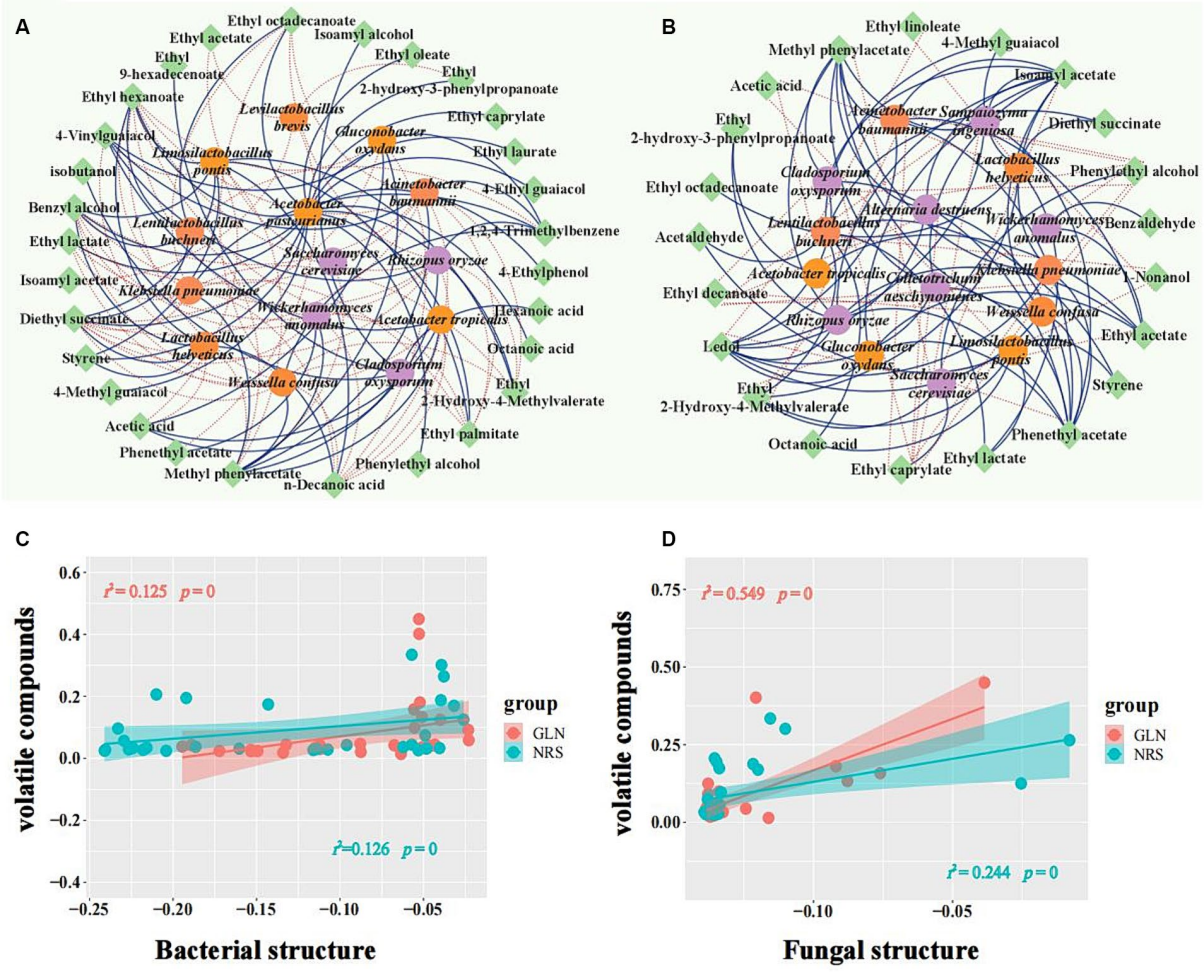
Relationship between microbial composition and volatile metabolites. A network of microorganisms related to major volatile compounds in GLN **(A)** and NRS **(B)** with a strong (Spearman’s |ρ| > 0.6) and significant (*p* < 0.05) correlation. Fungi, bacteria, and volatiles are represented by purple, orange, and green circle modules, respectively. Positive and negative correlations among microorganisms and volatiles are represented by solid and dotted edges, respectively. The relationship between volatile compounds and bacteria **(C)**, and fungi **(D)** was estimated with Pearson correlation. The microbial structure and content of volatile metabolites are indicated by the first axis of the PCA. The shaded area denotes 95% confidence intervals. Significance is represented by *p* < 0.05.

Flavor components are usually produced during fermentation by the microorganisms involved in the process. To determine the microbes that produce the main flavor components, co-occurrence network analysis was used to analyze the positive and negative correlations between the microbial taxa and major flavor components with relative contents over 0.1% (Figure 7). The results showed that *A. pasteurianus* exhibited a positive correlation with 17 flavor substances (10 ethyl ester compounds, 2 alcohols, 1 acid, 3 phenols, and 1 aromatic), thus indicating that this bacterium may have an important contribution to the formation of flavor substances in GLN. However, *La. buchneri* was significantly correlated with 11 flavor compounds, thus implying that it may play an important role in the formation of flavor compounds in NRS. Acetic acid was strongly positively associated with *A. tropicalis* (*ρ* > 0.8 and *p* < 0.01) and exhibited a significant negative association with *La. helveticus* and *La. pontis* in GLN, while it had an extremely significant negative correlation with *La. buchneri* and *La. pontis* (*ρ* > 0.8 and *p* < 0.001) in NRS. Ethyl acetate showed a strong positive association with *W. anomalus*, *R. oryzae*, *G. oxydans*, *A. baumannii*, and *A. tropicalis* (*ρ* > 0.8) and was negatively correlated with *S. cerevisiae* in NRS, while it only had a negative correlation with *S. cerevisiae* and *La. brevis* in GLN. Phenylethyl alcohol was positively correlated with *S. cerevisiae* and negatively correlated with 7 microbial species, including *W. confusa*, *K. pneumoniae*, and *A. baumannii*, etc., in NRS, while it only had a positive association with *A. pasteurianus* in GLN. In summary, 14 microorganisms and 29 flavor substances were found to form 117 co-occurrence networks in GLN, while 100 co-occurrence networks of 15 microorganisms with 21 flavor compounds were detected in NRS.

Furthermore, the correlation between the microbial community and the content of volatile compounds was calculated to evaluate the importance of biotic factors to volatile metabolites. The results showed that both the bacterial and fungal communities were significantly correlated with the volatile compounds (*p* < 0.05) in GLN and NRS (Figures 7C,D).

## Discussion

In traditional fermentation, the raw material plays a key role in the assembly of the core microbiota and the formation of volatile compounds. However, the effects of raw materials on the microbial composition and microbial function during Baijiu fermentation are still unclear. LFB is a valuable and convenient model for exploring the mechanism of microbial fermentation due to its short fermentation period and simple production process. Thus, the microbial composition and diversity and physicochemical dynamics during the fermentation of LFB produced from two types of sorghum varieties were investigated in this study.

We showed that the sorghum variety affected not only microbial succession but also microbial metabolism in Baijiu fermentation, based on both full-length amplicon sequencing and flavoromics. Previous studies have indicated that different sorghum varieties had significant discrepancy in their physicochemical constituents, such as amylopectin, tannins, and crude fat, which led to the different substrates available to microorganisms during the Baijiu fermentation (Wu et al., 2017). In this study, the glutinous sorghum had significantly higher amylopectin and tannin contents than non-glutinous sorghums (*p* < 0.05) (Supplementary Table S1), which was consistent with previous studies (Wu et al., 2017; Liu et al., 2023). Amylopectin, which has more short-chain branches, is easily decomposed into glucose by amylase; thus, the glutinous sorghum with a high content of amylopectin could provide more reducing sugar for microbial growth and metabolism at the beginning of fermentation (Wang et al., 2008; Xu et al., 2021). This may explain why the content of reducing sugar in GLN was higher than that in NRS in the early stages, while the residual starch content in GLN was lower during fermentation (Figures 1E,F). Similarly, previous studies have shown that the fermented grains of glutinous sorghum presented higher reducing sugar contents and lower starch contents than their non-glutinous counterparts during fermentation (Liu C. et al., 2021). In addition, tannins are also present at relatively high levels in glutinous sorghum and can affect the formation of flavor substances and microbial composition during Baijiu fermentation (Salami et al., 2018; Xu et al., 2018).

In the closed fermentation cellar, microenvironment plays a crucial role in regulating microbial succession and metabolism during fermentation. For instance, a variety of physicochemical factors, including acidity, amino nitrogen, residual starch, reducing sugar, and moisture contents, can regulate microbial dynamics during LFB brewing (Lin et al., 2022; Tang et al., 2022b). Our results indicated that moisture content, amino acid nitrogen, reducing sugar, residual starch, and total free amino acids were identified as the driving factors of the fungal and bacterial variation (Figures 6A,B). RDA further demonstrated that the microbial changes in GLN were positively related to moisture content, while amino acid nitrogen, residual starch, and total free amino acids were positively associated with the microbial community in NRS. These results were in agreement with the previous studies showing that differences in the composition of environmental factors produced by sorghum varieties had different effects on the microbial community (Liu et al., 2019; Wang et al., 2021). Moreover, compared with those in NRS, the microbial species in GLN responded more rapidly to environmental changes. Thus, sorghum varieties indirectly affected the microbial community by regulating the physicochemical properties of fermented grains.

Previous studies have suggested that the LFB fermentation process can be divided into two stages according to microbial changes in fermented grains (Shen et al., 2021; Lin et al., 2022). As illustrated in Figure 3, our current research also indicated that fungal and bacterial succession included two stages, and the microbial community changed the most after 2 days of fermentation, regardless of the variety of sorghum that was used. As fermentation proceeded, *Lactobacillus*, *Acetobacter*, and *Saccharomyces* became the dominant genera in the fermented grains in the middle and later stages. Further analysis revealed that the fungal community was more sensitive to changes in the fermentation environment due to rapid community structure changes after day 1 (Figure 3B), which was consistent with the findings of a previous study (Lin et al., 2022). Compared with the bacterial community, the fungal species composition showed less discrepancy between two varieties of sorghum in later fermentation, and anoxic and highly acidic fermentation environments promoted the formation of a fungal community that was mainly composed of *S. cerevisiae*. Besides, more differential species belonging to lactic acid bacteria were found in GLN, which correspondingly inhibited other bacteria by secreting lactic acid. In contrast, the differentially abundant strains in NRS were mainly non lactic acid bacteria (Figures 4A,B). *Lactobacillus* has been reported to be abundant in the final stage of the fermentation of different flavors of Baijiu and to greatly contribute to the production of flavor substances, such as lactic acid (Dong et al., 2020; Du et al., 2020; Ji et al., 2023; Li et al., 2023). Moreover, the rapid increase in *Lactobacillus* abundance during LFB fermentation can also inhibit unnecessary microbes by forming a highly acidic fermentation environment, thus leading to similar fungal communities in different sorghum varieties during the later stage of fermentation.

The microbial species involved in Baijiu fermentation determine the flavor composition and textures of the raw liquor. Microbial changes caused by the application of different varieties of sorghum eventually led to changes in the composition of flavor substances. In this study, the content of esters was higher in NRS than that in GLN (Figures 5A,B), which represented the majority of volatiles found in LFB fermentation samples, consistent with prior studies (Huang et al., 2020; Zhu et al., 2022). *W. anomalus* was reported to be conducive to the synthesis of ester compounds during Chinese Baijiu brewing, and the content of esters can be increased by adding *W. anomalus* strains (Fan et al., 2019; Wang et al., 2020). Figures 4C,D, 7B indicated that *W. anomalus* was a differential fungal specie and showed a strong positive association with ethyl acetate in NRS; thus, the higher biomass of *W. anomalus* possibly improved the production of esters in the fermented grains with NRS. Additionally, the content of ethyl acetate, which was the main aromatic substance in LFB, first increased, then decreased and remained relatively stable in the later stage of fermentation in the two grain varieties (Figure 5B). This may be the result of high oxygen content in the early stages of fermentation and low oxygen content in the middle and later stages of fermentation (Shen et al., 2021).

Furthermore, 8 volatiles were identified as being important differential substances in fermentation by using two sorghum varieties based on the PLS-DA and STAMP (Figures 5D,E); however, these compounds were not the major flavor substances in LFB. This indicated that sorghum variety not only caused changes in the content of the main aroma substances (such as ethyl acetate) but also regulated the composition of several micro-flavor components (such as 4-vinylguaiacol, ledol, and benzaldehyde). Among them, 4-vinylguaiacol is an important fragrance component in Baijiu, wine, and beer (Xu et al., 2020b; Wang et al., 2022) and has medicinal value because of its potential anticancer and antioxidant activities (Bortolomeazzi et al., 2007; Luo et al., 2021). In addition, 4-vinylguaiacol was more abundant in GLN (Figure 5D) and had a significant positive association with *R. oryzae*, *A. tropicalis*, and *G. oxydans* and a negative correlation with *La. helveticus* and *La. pontis* (Figure 7A). Multiple microorganisms, such as *Lactobacillus* spp., *Bacillus* spp., *Candida*, *Brettanomyce*s, and *Aspergillus* spp., have been reported to degrade ferulic acid into 4-vinylguaiacol (Suezawa and Suzuki, 2007; Xu et al., 2020a). Notably, *R. oryzae*, *A. tropicalis*, and *G. oxydans* were significantly differential and more abundant microbial species in GLN (Figure 4), likely resulting in higher 4-vinylguaiacol content in GLN samples. Thus, different sorghum varieties played important roles in the flavor component formation of LFB.

Some studies have demonstrated the key effect of microbial structure on volatile metabolites (Jia et al., 2020; Liu D. et al., 2021). Herein, we found that both fungi and bacteria were the main drivers of volatile metabolites, regardless of the sorghum varieties (Figures 7C,D). This study highlighted the idea that the structure of both fungi and bacteria played an important role in maintaining volatile metabolites during the LFB fermentation. Overall, different sorghum varieties led to differences in the succession of the microbial community and changes in environmental factors, thus ultimately resulting in differences in flavor compounds.

## Conclusion

To our knowledge, this is the first study to reveal the effect of sorghum varieties on microbial community and volatile compounds in the fermentation of LFB. In this study, the microbial composition and diversity, physicochemical dynamics, and flavor components during the fermentation of LFB produced from two types of sorghum varieties were investigated. Based on the microbial structure, aromatic compounds, and physicochemical factors, our research successfully elucidated why GLN and NRS exhibited distinct characteristics. NRS exhibited higher amino acid nitrogen, residual starch, and total free amino acids contents, which were the main driving factors for the microbial community succession of the fermented grains in NRS. Higher bacteria α-diversity and more differential bacterial species were detected in GLN, while a higher fungal α-diversity and more differential fungal species were detected in NRS. In addition, the relative contents of volatiles in NRS were relatively higher than those in GLN, and 8 flavor compounds were identified as being important differential substances in the fermented grains of two sorghum varieties. The findings can provide guidance for selecting brewing materials and optimizing fermentation parameters to improve the quality of Baijiu.

## Data availability statement

The datasets presented in this study can be found in online repositories. The names of the repository/repositories and accession number(s) can be found in the article/Supplementary material.

## Author contributions

JT: Conceptualization, Funding acquisition, Investigation, Methodology, Writing – original draft, Writing – review & editing. BL: Investigation, Methodology, Writing – original draft. YS: Data curation, Methodology, Writing – original draft. SR: Data curation, Methodology, Writing – original draft. WJ: Data curation, Visualization, Writing – original draft. QL: Investigation, Methodology, Writing – original draft. LZ: Investigation, Methodology, Writing – original draft. RL: Investigation, Methodology, Writing – original draft. QY: Funding acquisition, Resources, Supervision, Writing – review & editing. HD: Investigation, Methodology, Resources, Writing – review & editing. SY: Resources, Supervision, Writing – review & editing. QS: Resources, Supervision, Writing – review & editing. SC: Conceptualization, Funding acquisition, Supervision, Validation, Writing – review & editing.
