# Supplementary material for: Effects of sorghum varieties on microbial communities and volatile compounds in the fermentation of light-flavor Baijiu
Source: Front Microbiol. 2024 Jul 31;15:1421928. doi: 10.3389/fmicb.2024.1421928 (PMC11322492; doi:10.3389/fmicb.2024.1421928)
Supplement: Supplementary file 1 [file Data_Sheet_1.docx]

Effects of sorghum varieties on microbial communities and volatile compounds in the fermentation of light-flavor Baijiu

**Jie Tang^1^, Bin Lin^1^, Yimin Shan^1^, Song Ruan^1^, Wei Jiang^1^, Qun Li^1^, Liping Zhu^1^, Rui Li^1^, Qiang Yang^1^, Hai Du^2^, Shengzhi Yang^1^, Qi Sun^1^, Shenxi Chen^1^***

^1^*Hubei key Laboratory of Quality and Safety of Traditional Chinese Medicine Health Food, Jing Brand Research Institute, Jing Brand Co., Ltd., Daye, China*

^2^*Lab of Brewing Microbiology and Applied Enzymology, Key Laboratory of Industrial Biotechnology of Ministry of Education, School of Biotechnology, Jiangnan University, Wuxi, China*

**Contents** **Page**

1. **Figure S1.** Comparative analysis of physicochemical properties in GLN and NRS

3

1. **Figure S2.** Rarefaction curves of bacterial (a) and fungal (b) community in GLN and NRS 4
2. **Figure S3.** Comparative analysis of microbial community α-diversity indices in GLN and NRS 5
3. **Table S1** Characteristics of chemical compositions of glutinous and nonglutinous sorghum cultivars 6
4. **Table S2** Correlation of microorganisms and physicochemical properties in GLN

7

1. **Table S3** Correlation of microorganisms and physicochemical properties in NRS

9

**A**


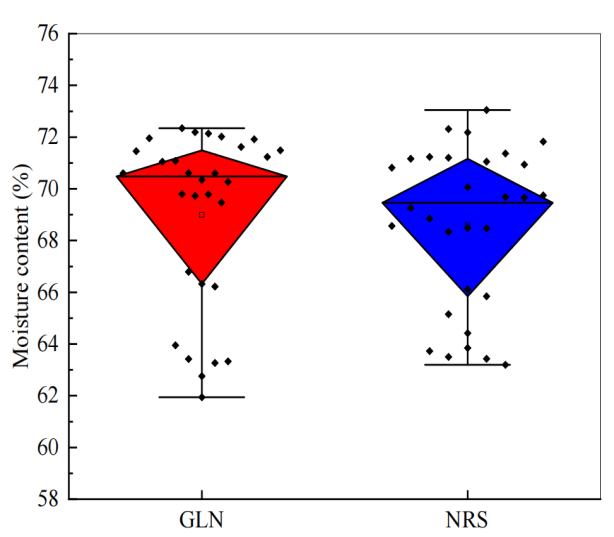


**B**


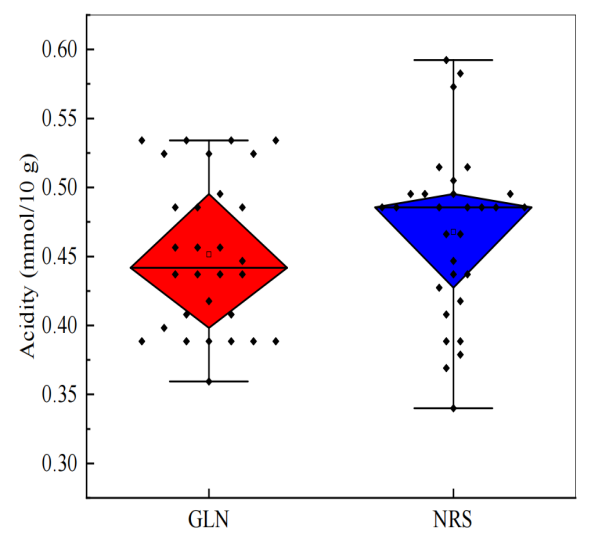


**C**


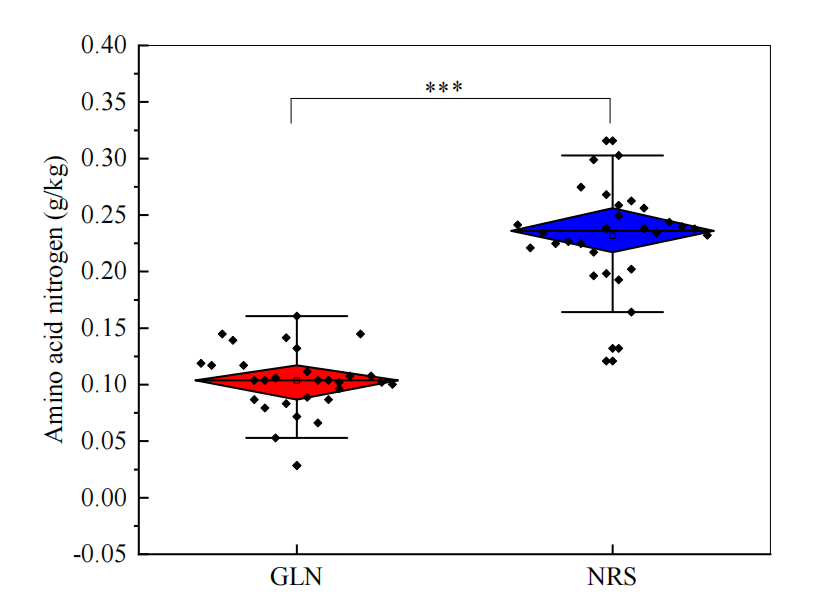


**D**


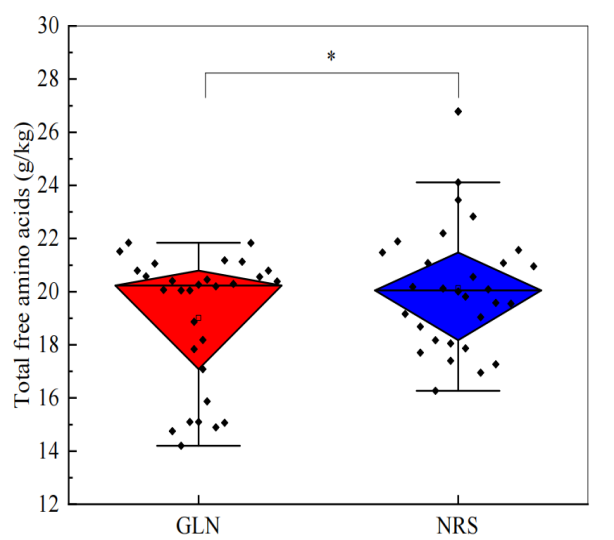

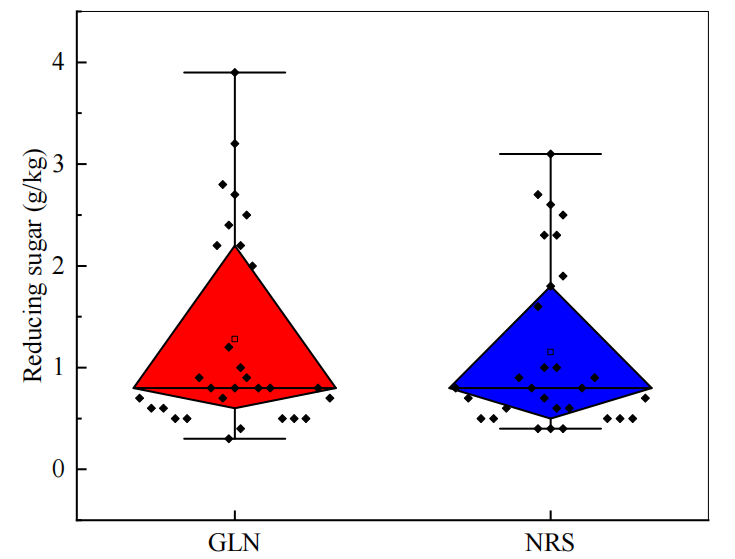


**E**


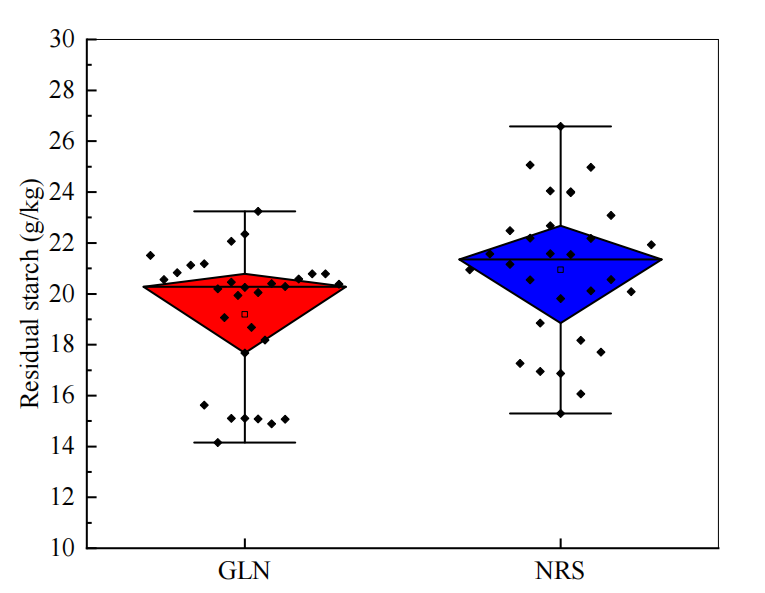


**F**

**Figure S1.** Comparative analysis of physicochemical properties in GLN and NRS. (**A**) Moisture content. (**B**) Acidity. (**C**) Amino acid nitrogen. (**D**) Total free amino acids. (**E**) Reducing sugar. (**F**) Residual starch. *: *p* < 0.05; ***: *p* < 0.001.


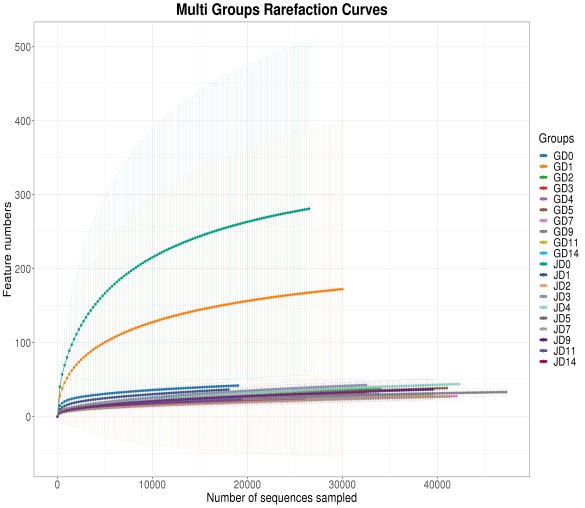


**B**

**A**


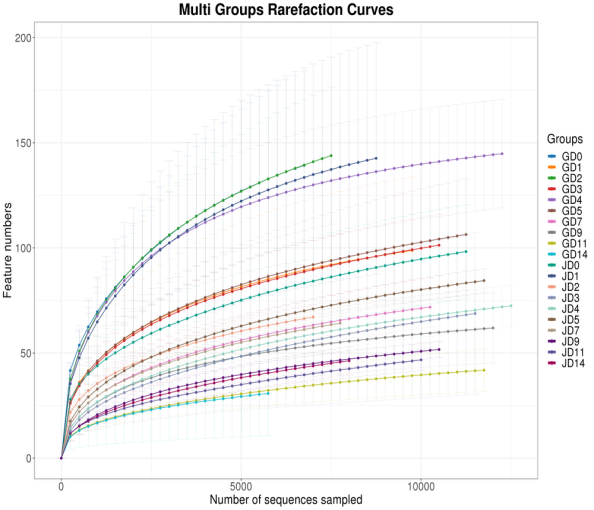


**Figure S2.** Rarefaction curves of bacterial (**A**) and fungal (**B**) community in GLN and NRS.

**B**


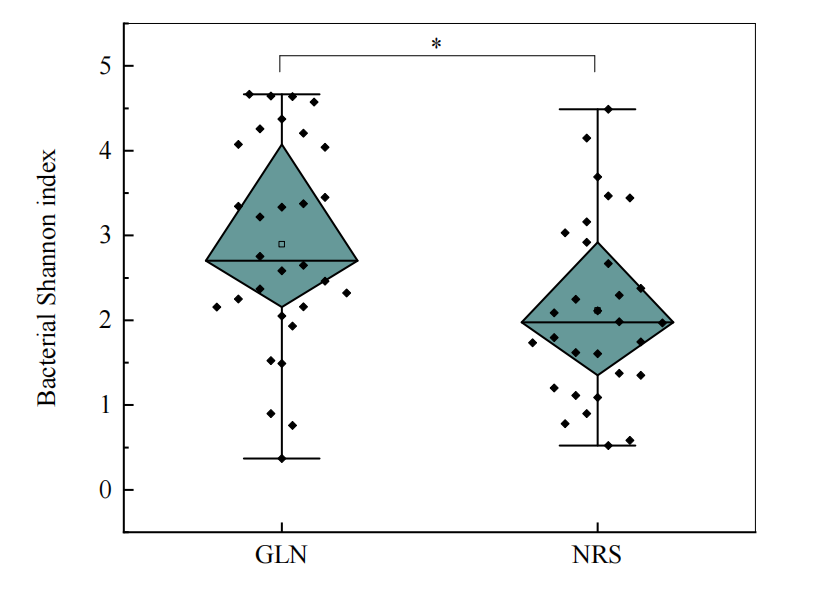


**A**


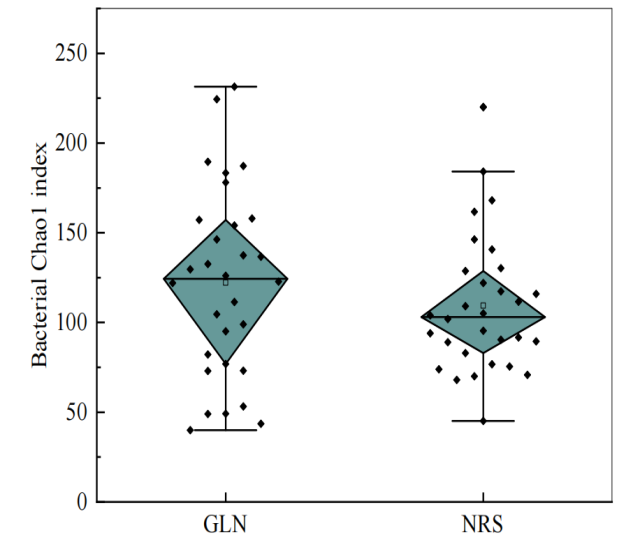

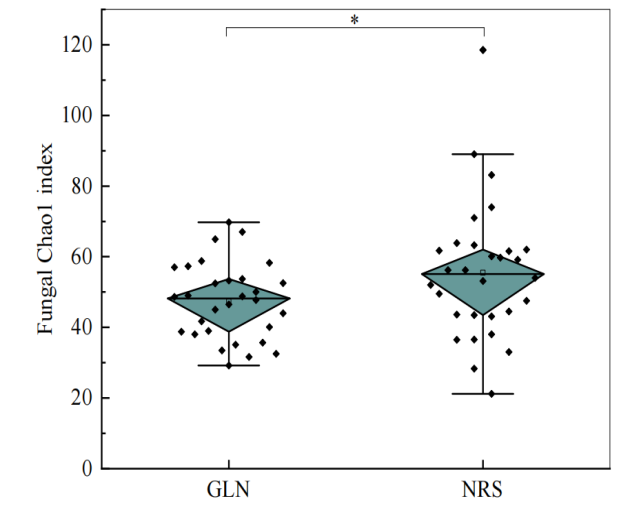


**C**


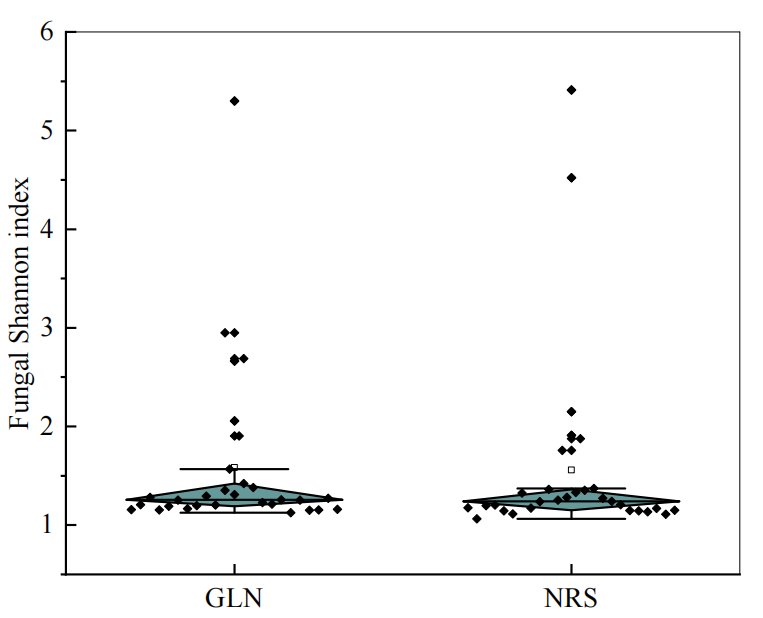


**D**

**Figure S3.** Comparative analysis of microbial community α-diversity indices in GLN and NRS. (**A**) Bacteria Chao1 indices. (**B**) Bacteria Shannon indices. (**C**) Fungi Chao1 indices. (**D**) Fungi Shannon indices. *: *p* < 0.05.

**Table S1** Characteristics of chemical compositions of glutinous and non-glutinous sorghum cultivars (g/kg dry weight)

| **Cultivars** | **Total starch** | **Amylose** | **Amylopectin** | **Crude protein** | **Crude fiber** | **Tannin** |
| --- | --- | --- | --- | --- | --- | --- |
| GLN | 77.92±2.01 a | ND | 77.92±2.01 a | 9.47±0.57 a | 3.63±0.32 a | 1.13±0.30 a |
| NRS | 68.62±0.83 b | 8.02±2.42 a | 60.50±2.28 b | 10.47±1.22 a | 3.80±0.58 a | 0.77±0.09 b |

Values with the different letter in a column represented significant differences at *p* < 0.05. Results were given as averages of nine replicates ± standard deviation.

**Table S2** Correlation of microorganisms and physicochemical properties in GLN.

| **Species** | **Physicochemical properties** | **ρ** | ***p*** | **Species** | **Physicochemical properties** | **ρ** | ***p*** |
| --- | --- | --- | --- | --- | --- | --- | --- |
| *Acetobacter tropicalis* | Moisture content | -0.68 | 0.035 | *Gluconobacter oxydans* | Reducing sugar | 0.70 | 0.031 |
| *Acetobacter tropicalis* | Acidity | -0.89 | 0.001 | *Gluconobacter oxydans* | Residual starch | 0.81 | 0.008 |
| *Acetobacter tropicalis* | Amino acid nitrogen | -0.70 | 0.031 | *Klebsiella pneumoniae* | Moisture content | -0.92 | 0.000 |
| *Acetobacter tropicalis* | Reducing sugar | 0.65 | 0.049 | *Klebsiella pneumoniae* | Acidity | -0.67 | 0.035 |
| *Acetobacter tropicalis* | Residual starch | 0.76 | 0.016 | *Klebsiella pneumoniae* | Total free amino acids | -0.72 | 0.024 |
| *Acetobacter pasteurianus* | Residual starch | -0.66 | 0.044 | *Klebsiella pneumoniae* | Reducing sugar | 0.90 | 0.001 |
| *Acinetobacter baumannii* | Moisture content | -0.93 | 0.000 | *Klebsiella pneumoniae* | Residual starch | 0.89 | 0.001 |
| *Acinetobacter baumannii* | Acidity | -0.77 | 0.010 | *Lactobacillus helveticus* | Reducing sugar | -0.84 | 0.004 |
| *Acinetobacter baumannii* | Total free amino acids | -0.71 | 0.021 | *Lactobacillus helveticus* | Residual starch | -0.81 | 0.008 |
| *Acinetobacter baumannii* | Reducing sugar | 0.89 | 0.001 | *Lactobacillus helveticus* | Moisture content | 0.88 | 0.002 |
| *Acinetobacter baumannii* | Residual starch | 0.98 | 0.000 | *Lactobacillus helveticus* | Acidity | 0.74 | 0.014 |
| *Cladosporium oxysporum* | Acidity | -0.69 | 0.029 | *Lactobacillus helveticus* | Amino acid nitrogen | 0.66 | 0.044 |
| *Gluconobacter oxydans* | Moisture content | -0.73 | 0.021 | *Lentilactobacillus buchneri* | Reducing sugar | -0.81 | 0.008 |
| *Gluconobacter oxydans* | Acidity | -0.87 | 0.001 | *Lentilactobacillus buchneri* | Residual starch | -0.96 | 0.000 |
| *Gluconobacter oxydans* | Amino acid nitrogen | -0.67 | 0.039 | *Lentilactobacillus buchneri* | Moisture content | 0.87 | 0.003 |
| *Limosilactobacillus pontis* | Reducing sugar | -0.85 | 0.004 | *Lentilactobacillus buchneri* | Acidity | 0.77 | 0.010 |
| *Limosilactobacillus pontis* | Residual starch | -0.84 | 0.004 | *Weissella confusa* | Moisture content | -0.90 | 0.001 |
| *Limosilactobacillus pontis* | Moisture content | 0.87 | 0.003 | *Weissella confusa* | Acidity | -0.78 | 0.007 |
| *Limosilactobacillus pontis* | Acidity | 0.74 | 0.014 | *Weissella confusa* | Total free amino acids | -0.65 | 0.049 |
| *Limosilactobacillus pontis* | Total free amino acids | 0.67 | 0.039 | *Weissella confusa* | Reducing sugar | 0.87 | 0.003 |
| *Rhizopus oryzae* | Moisture content | -0.92 | 0.000 | *Weissella confusa* | Residual starch | 0.98 | 0.000 |
| *Rhizopus oryzae* | Acidity | -0.77 | 0.009 | *Wickerhamomyces anomalus* | Moisture content | -0.84 | 0.004 |
| *Rhizopus oryzae* | Total free amino acids | -0.67 | 0.039 | *Wickerhamomyces anomalus* | Acidity | -0.73 | 0.017 |
| *Rhizopus oryzae* | Reducing sugar | 0.88 | 0.002 | *Wickerhamomyces anomalus* | Total free amino acids | -0.75 | 0.018 |
| *Rhizopus oryzae* | Residual starch | 0.99 | 0.000 | *Wickerhamomyces anomalus* | Reducing sugar | 0.78 | 0.012 |
| *Saccharomyces cerevisiae* | Reducing sugar | -0.75 | 0.018 | *Wickerhamomyces anomalus* | Residual starch | 0.88 | 0.002 |
| *Saccharomyces cerevisiae* | Residual starch | -0.70 | 0.031 | *Saccharomycodes ludwigii* | Residual starch | -0.83 | 0.006 |
| *Saccharomyces cerevisiae* | Moisture content | 0.71 | 0.028 | *Saccharomycodes ludwigii* | Moisture content | 0.81 | 0.008 |
| *Saccharomycodes ludwigii* | Reducing sugar | -0.75 | 0.018 |  |  |  |  |

**Table S3** Correlation of microorganisms and physicochemical properties in NRS.

| **Species** | **Physicochemical properties** | **ρ** | ***p*** | **Species** | **Physicochemical properties** | **ρ** | ***p*** |
| --- | --- | --- | --- | --- | --- | --- | --- |
| *Acetobacter tropicalis* | Moisture content | -0.90 | 0.001 | *Klebsiella pneumoniae* | Total free amino acids | -0.87 | 0.003 |
| *Acetobacter tropicalis* | Total free amino acids | -0.95 | 0.000 | *Klebsiella pneumoniae* | Reducing sugar | 0.74 | 0.014 |
| *Acetobacter tropicalis* | Reducing sugar | 0.81 | 0.005 | *Klebsiella pneumoniae* | Residual starch | 0.87 | 0.003 |
| *Acetobacter tropicalis* | Residual starch | 0.83 | 0.006 | *Lactobacillus helveticus* | Residual starch | -0.87 | 0.003 |
| *Acinetobacter baumannii* | Moisture content | -0.94 | 0.000 | *Lactobacillus helveticus* | Moisture content | 0.92 | 0.000 |
| *Acinetobacter baumannii* | Total free amino acids | -0.96 | 0.000 | *Lactobacillus helveticus* | Total free amino acids | 0.90 | 0.001 |
| *Acinetobacter baumannii* | Reducing sugar | 0.84 | 0.002 | *Lentilactobacillus buchneri* | Reducing sugar | -0.76 | 0.011 |
| *Acinetobacter baumannii* | Residual starch | 0.89 | 0.001 | *Lentilactobacillus buchneri* | Residual starch | -0.89 | 0.001 |
| *Gluconobacter oxydans* | Moisture content | -0.92 | 0.000 | *Lentilactobacillus buchneri* | Moisture content | 0.98 | 0.000 |
| *Gluconobacter oxydans* | Amino acid nitrogen | -0.65 | 0.049 | *Lentilactobacillus buchneri* | Total free amino acids | 0.94 | 0.000 |
| *Gluconobacter oxydans* | Total free amino acids | -0.94 | 0.000 | *Limosilactobacillus pontis* | Reducing sugar | -0.78 | 0.007 |
| *Gluconobacter oxydans* | Reducing sugar | 0.82 | 0.004 | *Limosilactobacillus pontis* | Residual starch | -0.89 | 0.001 |
| *Gluconobacter oxydans* | Residual starch | 0.85 | 0.004 | *Limosilactobacillus pontis* | Moisture content | 0.85 | 0.004 |
| *Klebsiella pneumoniae* | Moisture content | -0.84 | 0.004 | *Limosilactobacillus pontis* | Total free amino acids | 0.82 | 0.007 |
| *Rhizopus oryzae* | Moisture content | -0.92 | 0.000 | *Saccharomyces cerevisiae* | Total free amino acids | 0.89 | 0.001 |
| *Rhizopus oryzae* | Total free amino acids | -0.88 | 0.002 | *Saccharomycodes ludwigii* | Amino acid nitrogen | -0.71 | 0.028 |
| *Rhizopus oryzae* | Reducing sugar | 0.85 | 0.002 | *Weissella confusa* | Moisture content | -0.88 | 0.002 |
| *Rhizopus oryzae* | Residual starch | 0.82 | 0.007 | *Weissella confusa* | Total free amino acids | -0.87 | 0.003 |
| *Saccharomyces cerevisiae* | Reducing sugar | -0.76 | 0.011 | *Weissella confusa* | Reducing sugar | 0.92 | 0.000 |
| *Saccharomyces cerevisiae* | Residual starch | -0.82 | 0.007 | *Weissella confusa* | Residual starch | 0.95 | 0.000 |
| *Saccharomyces cerevisiae* | Moisture content | 0.95 | 0.000 | *Wickerhamomyces anomalus* | Amino acid nitrogen | -0.75 | 0.018 |
